# Small-cell carcinoma of the gastrointestinal tract: a retrospective study of 64 cases

**DOI:** 10.1038/sj.bjc.6601758

**Published:** 2004-04-13

**Authors:** B Brenner, M A Shah, M Gonen, D S Klimstra, J Shia, D P Kelsen

**Affiliations:** 1 The Gastrointestinal Oncology Service, Department of Medicine, Memorial Sloan Kettering Cancer Center, New York, NY 10021, USA; 2 The Weill School of Medicine, Cornell University, New York, NY 10021, USA; 3 The Department of Epidemiology and Biostatistics, Memorial Sloan Kettering Cancer Center, New York, NY 10021, USA; 4 The Department of Pathology, Memorial Sloan Kettering Cancer Center, New York, NY 10021, USA

**Keywords:** small-cell carcinoma, the gastrointestinal tract

## Abstract

Small-cell carcinoma (SmCC) of the gastrointestinal tract is a very rare and aggressive malignancy. To better define its clinicopathological features, the records of all patients with this disease seen at Memorial Sloan Kettering Cancer Center between 1980 and 2002 (*n*=64) were reviewed. The most common primary tumour locations were in the large bowel and oesophagus. Predisposing medical conditions for non-small-cell cancers, positive family cancer history, and metachronous tumours were common. In all, 37% had mixed tumour histology and 48% presented with extensive disease, according to the Veterans' Administration Lung Study group (VALSG) staging system used for small-cell lung cancer. Treatment outcome in limited disease (LD) suggested a role for surgery and chemotherapy. Platinum-based regimens resulted in a 50% response rate. The 2-year survival was 23% and two prognostic factors were identified, the extent of disease according to the VALSG system (*P*<0.01) and TNM stage (*P*=0.03). Anatomic location had no clinical impact. In conclusion, SmCC from various gastrointestinal sites can be viewed as one clinical entity. Mixed tumour histology is common and may affect therapy. Surgery, combined with chemotherapy, should be considered for LD. The value of the VALSG system was implied and possible differences from small-cell lung cancer were noted.

Small-cell carcinoma (SmCC) of the gastrointestinal tract (GIT) is a highly aggressive and lethal neoplasm. Initially described in the oesophagus, by Mckeown (1952) in (Mckeown, 1952), the disease has been since reported to arise in all parts of the gastrointestinal system (Toker, 1974; Wick *et al*, 1987; Matsui *et al*, 1991; Chetty *et al*, 1993; Hoff and Pazdur, 1999; Maitra *et al*, 2001). Small-cell carcinoma of the GIT is very uncommon, with a total of approximately 544 cases having been reported in the English literature. In light of its rarity, and the fact that it was so far studied from an organ-centred perspective, data with regard to this entity is extremely limited. Owing to the paucity of information and its resemblance to the well-recognised primary SmCC of the lung (Kelsen *et al*, 1980; Fer *et al*, 1981; Ibrahim *et al*, 1984), the disease is usually managed as the latter (Nichols and Kelsen, 1989; Huncharek and Muscat, 1995). However, the extent of the similarity between the two entities has been recently questioned; data from a literature review that we have performed (Brenner *et al*, 2004), as well as from other reports, suggest some differences between the two (Nishimaki *et al*, 1997; Arai and Matsuda, 1998; Takubo *et al*, 1999; Maitra *et al*, 2001). In order to better define the clinical features of the disease, as well the impact of various treatment modalities, we conducted a retrospective analysis of all patients with gastrointestinal (GI) SmCC who were seen at Memorial Sloan Kettering Cancer Center between 1980 and 2002. Doing so, we intended to examine the significance of the specific site within the GIT from which these tumours arise and whether they can indeed be studied as one clinicopathological entity.

## PATIENTS AND METHODS

The medical records of all patients with a histologically proven diagnosis of SmCC of the GIT seen at Memorial Sloan Kettering Cancer Center during the period 1980–2002 were reviewed. The following data were collected for each patient: age, gender, ethnicity, past medical and family histories, smoking and alcohol habits, clinical presentation, anatomic location, stage, treatment details and clinical course. The histological diagnosis of SmCC was confirmed by the Memorial Sloan Kettering Cancer Center pathology department in all cases. Whenever pathology slides were available, this diagnosis was also retrospectively corroborated by two independent pathologists (DSK, JS). Staging work-up differed according to the primary site. However, it generally included gastrointestinal endoscopy (colonoscopy or oesophagogastroscopy) and computed tomography scan of the chest, abdomen and pelvis. Additional tests that were commonly used were bronchoscopy, bone scan and bone marrow biopsy. In recent years, positron emission tomography and magnetic resonance imaging were also used occasionally.

In the absence of a specific staging system established for SmCC of the GIT, the stage of the disease is presented according to two staging systems, which are being used in parallel in clinical practice. The first is the 2002 American Joint Committee on Cancer (AJCC) TNM staging system for adenocarcinomas and squamous cell carcinomas of each of the affected organs (American Joint Committee on Cancer, 2001) The second is the system introduced by the Veterans' Administration Lung Study group (VALSG) for primary SmCC of the lung (Zelen, 1973). This system consists of two staging categories, limited and extensive disease (ED). Limited disease (LD) is defined as a tumour contained within a localised anatomic region, with or without regional lymph node involvement. Extensive disease is defined as a tumour outside the locoregional boundaries (Van Der Gaast *et al*, 1990).

Survival was analysed as disease-specific survival, calculated as the time from diagnosis to death or to last follow-up date. This is subject to length-biased sampling (Simon, 1980), but the time from diagnosis to treatment was less than 30 days for 90% of the patients, so we assumed the bias to be small. Patients dying of causes other than SmCC of the GIT, including second malignancies, were censored. Survival was estimated by the Kaplan–Meier method (Kaplan and Meier, 1958). Analysis of prognostic factors for survival included only those patients with at least 2 months of follow-up. Comparisons of survival estimates between subgroups according to clinical variables were performed using the log-rank test (Mantel, 1966). Cox's proportional-hazard regression models were applied for multivariate analysis and to obtain hazard ratios and 95% confidence intervals (CI) (Cox, 1972). *P*-values less or equal to 0.05 were considered statistically significant.

## RESULTS

### Patients and tumours

In all, 64 patients with SmCC of the GIT were traced through the Memorial Sloan Kettering Cancer Center database. This represented less than 1% of all patients with GI malignancies seen in the cancer centre during the period from 1980 to 2002. The histological diagnosis was based on biopsy material in 33 patients and on the surgical specimen in 31. In 53 cases (83%), the pathology slides were retrospectively reviewed by one of the authors (DSK, JS) and the diagnosis of SmCC was confirmed in all cases. None of the patients had evidence of a primary tumour in the lung.

The clinical characteristics at presentation are outlined in Table 1

**Table 1:** Clinical features at presentation (*n*=64)

	**Number^a^**	**Valid %**
*Age (years)*		
Median (range)	61 (37–86)	
		
*KPS*		
Median (range)	80 (40–90)	
		
*Gender*		
Male	31	48.4
Female	33	51.6
		
*Ethnicity*		
Caucasian	53	84.1
Black	5	7.9
Hispanic	3	4.8
Asian	2	3.2
		
*Anatomic location*		
Oesophagus^b^	19	29.7
Stomach	5	7.8
Gallbladder	5	7.8
Pancreas	6	9.4
Small bowel	2	3.1
Colon	13	20.3
Rectum	12	18.8
		
*Second malignancies*		
Yes	11	20.4
No	43	79.6
		
*Predisposing medical conditions*^c^		
Yes	25	46.3
None	29	53.7
		
*Family history*		
GI malignancies	9	30.0
Other malignancies	6	20.0
None	15	50.0
		
*Presenting symptom*		
Weight loss	15	51.7
Pain	18	47.4
Obstruction	15	38.5
Bleeding	8	20.0
Mass	5	12.2

KPS=Karnofsky performance status; SmCC=small-cell carcinoma.

a Data were missing with regard to ethnicity (one patient), other malignancies (10), predisposing medical condition (10), family history (34), weight loss (35), pain (26), obstruction (25), bleeding (24) and mass (23).

b Including four patients with tumours in gastro-oesophageal junction.

c Patients with at least one predisposing medical conditions for the development of non-SmCC tumours in the same organ: smoking (oesophageal, gastric and pancreatic tumours), alcohol use (oesophagus), gastro-oesophageal reflux disease (oesophagus), biliary disease (gallbladder), pancreatitis (pancreas), polyps (large bowel) and homosexuality (anus).s

. The median age was 61 years, with almost equal gender distribution. The median Karnofsky performance status (KPS) was 80% (range, 40–90%). The vast majority of patients (84%) were Caucasians. The most common primary sites were the large bowel (39%), followed by the oesophagus (30%). In all, 11 patients (20%) had 13 metachronous second malignancies. Six of these patients had other GI malignancies, mostly colorectal cancer. Of the 54 patients with available data, 25 (46%) had recognised predisposing medical conditions for the development of non-SmCC tumours in the same organ. Notably, 12 of 21 patients (57%) with colorectal SmCC had either colonic villous adenomas (11 patients) or ulcerative colitis (one). Eight of 18 patients (44%) with oesophageal SmCC had at least one predisposing factor (smoking, alcohol use or gastro-oesophageal reflux disease) and one of the two patients with anal SmCC was homosexual and had HIV. Half of the 30 patients with available information had a positive family history for malignancies, primarily gastrointestinal in origin. While data with regard to presenting symptoms were clearly specified in approximately only half of all patients, they still demonstrate a clear pattern; the most common presentations were weight loss (52% of patients) and pain (47%), followed by obstructive symptoms (38%). Bleeding symptoms (20%) and a presentation with a mass (12%) were less common. One patient presented with a full-blown Cushing syndrome and one was diagnosed during pregnancy.

Tumour characteristics are summarised in Table 2

**Table 2:** Tumour characteristics (*n*=64)

	**Number^a^**	**Valid %**
*Other histological component*		
Adenocarcinoma	13	21.0
Squamous cell carcinoma	9	14.5
Anaplastic carcinoma	1	1.6
Pure SmCC	39	62.9
		
*T stage*		
I	5	9.8
II	9	17.7
III	25	49.0
IV	12	23.5
		
*N status*		
N+	34	66.7
N0	17	33.3
		
*M status*		
M1	35	55.6
M0	28	44.4
		
*Sites of metastases at presentation*^b^		
Liver	22	37.3
Lung	6	10.5
Adrenal	1	1.8
Peritoneum	9	15.2
Soft tissue	7	12.3
Bone	6	10.3
Brain	1	1.8
		
*Number of metastatic sites*		
0	28	44.4
1	11	17.5
2	13	20.6
⩾3	11	17.5
		
*TNM stage*^c^		
I	4	6.8
II	9	15.2
III	11	18.6
IV	35	59.3
		
*Extent*		
Limited	33	52.4
Extensive	30	47.6
SmCC=small-cell carcinoma.		

a Data were missing with regard to other histological component (2 patients), T stage (13), N status (13), M status (1), number of metastatic sites (1), TNM stage (5) and extent of disease (1).

b Involvement of the following sites was not specified: liver (5 patients), lung (7), adrenal (8), peritoneum (5), soft tissue (7), bone (6) and brain (8).

c According to the 2002 AJCC staging system.

. The most common site for metastatic involvement at presentation was the liver. Other common sites were the peritoneum, soft tissues, lungs and bones. Overall, 78% of patients had a disease classified as TNM stage III or IV and 48% had ED.

### Treatment

Treatment results were analysed using the VALSG (LD *vs* ED) staging system, for purposes of uniformity among the different tumour sites and because this system was more commonly used in the management of these patients. Treatment information was available for 52 patients, 26 with LD and 26 with ED. The primary treatment approach for LD varied; surgery, chemotherapy and radiotherapy were all used, either alone or as part of a variety of combined modality treatment schemes. On the other hand, in 20 of the 26 patients (77%) with ED, primary treatment consisted exclusively of chemotherapy. The other patients were treated by palliative surgery (three patients), radiotherapy (one) or by supportive care (two).

Of the 17 patients with LD who received primary treatment with surgery, alone or with other treatment modalities, three patients, two of whom were treated by surgery alone, are still alive with no evidence of disease (ANED) 15, 94 and 99 months from diagnosis. One patient died with no evidence of disease almost 9 years from diagnosis. Three of the operated patients died from postoperative complications; in seven of the remaining 14 patients (50%), locoregional control was preserved. Eight patients received radiotherapy as part of their primary treatment. At the time of the analysis, only one of these remained free of disease recurrence. Locoregional failure was common; six of seven patients for whom such data were available had locoregional progression after radiation. In total, 16 patients with LD received chemotherapy, 12 of whom received it in conjunction with either surgery or radiotherapy. Of these, two remained ANED for at least 64 and 94 months and one expired with no evidence of recurrence after almost 9 years. Locoregional control was preserved in five (31%) of these patients.

Overall, for both LD and ED, 36 patients received chemotherapy, with or without other modalities, as their primary treatment. Half the patients were treated by a combination of etoposide and platinum compound. The rest of the patients were treated by a variety of regimens, such as 5-fluorouracil/leucovorin and CAV (cyclophosphamide, doxorubicin and either vincristine or etoposide). A total of of 14 patients were nonevaluable for response; they either received chemotherapy with concurrent radiotherapy or in the adjuvant setting. Of the 22 evaluable patients, two complete (9%) and six partial responses (27%) were observed, with an overall objective response rate of 36%. The median duration of response was 8 months (range, 4–16 months). With small absolute numbers, regimens that seemed to have been associated with significant clinical activity, in the order of 50% objective response rate, are the platinum-based and the CAV regimens. Data on newer agents, such as paclitaxel and irinotecan, were very limited.

### Clinical course

**Figure 1:**
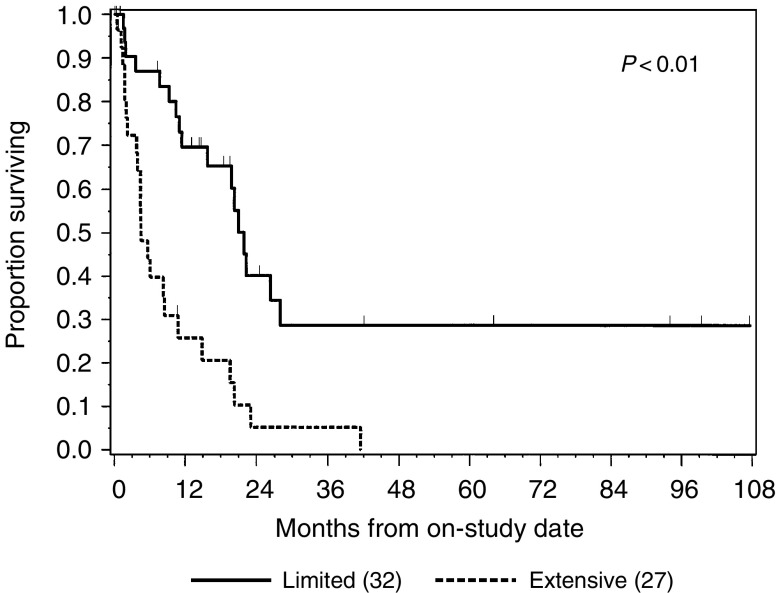
Disease-specific survival by the extent of disease (*n*=59). Within parenthesis is the number of patients in each subgroup.

At a median follow-up of 13 months (range, 1–107 months), six of the 64 patients (9%) were ANED, 16 patients (25%) were alive with disease, one patient (2%) died without any evidence of disease and 41 patients (64%) died of disease. The 1- and 2-year survival rates for the entire group were 49 and 23%, respectively. Four patients (6%) survived more than 5 years. The median survival was 11 months (range, 1–107 months) (Figure 1

).

Of the 48 patients with known progression status and at least 1 month of follow-up, progression was documented in 43 patients (90%). First progression was locoregional in 11 patients (26%), distant in 12 (28%), mixed in 10 (23%) and unspecified in 10 (23%). Data of the specific location of metastases, available for 80% of the patients, demonstrated a distinct pattern. The most common sites of metastases, at any time during the follow-up, were lymph nodes (75%) and liver (53%), followed by the peritoneum (28%), soft tissues (25%) and lungs (22%). Bone (17%), brain and adrenals (10% each) were less commonly involved.

### Prognostic factors

The results of a univariate analysis of various patient and tumour variables are depicted in Table 3

**Table 3:** Survival by patient and tumour characteristics

	**Univariate analysis**			
	**Overall survival**	**1-year survival**	**Median survival, months (95% CI)**	***P*-value**
*Age (years)*				
⩽60 (*n*=29)	45%	63%	20.3 (10.8–22.3)	0.18
>60 (*n*=31)	32%	43%	10.4 (5.6–21.9)	
				
*Gender*				
Male (*n*=29)	41%	34%	10.4 (7.6–19.8)	0.52
Female (*n*=31)	35%	68%	203 (14.9–23.1)	
				
*KPS*				
⩽80 (*n*=22)	18%	27%	7.0 (4.4–11.4)	<0.01
>80 (*n*=18)	53%	82%	20.3 (15.8–NR)	
				
*Anatomic location*^a^				
Upper GIT (*n*=24)	25%	43%	9.2 (6.1–22.3)	0.37
Lower GIT (*n*=24)	54%	67%	19.6 (11.4–NR)	
Other (*n*=12)	33%	48%	10.8 (4.4–NR)	
				
*Weight loss*				
Yes (*n*=14)	36%	36%	7.6 (3.9–15.8)	<0.01
None (*n*=14)	43%	84%	21.9 (14.9–NR)	
				
*Obstruction*				
Yes (*n*=14)	36%	55%	15.8 (8.5–28.0)	0.51
None (*n*=23)	39%	63%	19.8 (10.8–26.3)	
				
*Other histological component*				
Yes (*n*=21)	62%	50%	11.4 (7.6–21.0)	0.14
None (*n*=37)	27%	61%	21.1 (4.4–NR)	
				
*T stage*				
I (*n*=5)	40%	80%	21.9 (2.0–NR)	<0.01
II (*n*=8)	25%	63%	21.3 (7.6–NR)	
III (*n*=22)	64%	71%	26.3 (11.0–NR)	
IV (*n*=12)	8%	27%	4.4 (3.8–14.9)	
				
*N status*				
N+ (*n*=29)	28%	33%	7.6 (4.4–14.9)	0.02
N0 (*n*=17)	41%	88%	22.0 (19.8–28.0)	
				
*M status*				
M1 (*n*=32)	28%	31%	8.3 (4.4–11.0)	<0.01
M0 (*n*=27)	52%	79%	22.0 (19.8–28.0)	
				
*TNM stage*				
I (*n*=4)	25%	75%	19.8 (2.0–22.0)	0.04
II (*n*=9)	44%	89%	26.3 (20.3–NR)	
III (*n*=10)	60%	75%	28.0 (10.4–NR)	
IV (*n*=32)	28%	31%	8.3 (4.4–11.0)	
				
*Extent*				
Limited (*n*=32)	50%	72%	21.9 (15.8–NR)	<0.01
Extensive (*n*=27)	26%	29%	5.8 (4.4–10.8)	

NR=not reached.

a Upper GIT=oesophagus and stomach; lower GIT=large bowel and anus; other=gallbladder, pancreas and small bowel; KPS=Karnofsky performance status; SmCC=small-cell carcinoma.

. Several factors were found to correlate with patient outcome: performance status, weight loss, T stage, N and M status, TNM stage and extent of disease (LD *vs* ED). The extent of disease had a strong impact on survival; patients with LD had 72% 1-year survival as compared with those with ED, who had 29% 1-year survival (*P*<0.01) (Figure 1). Patients with TNM stages I, II and III had similar survival profiles and their prognosis was better than stage IV patients (*P*=0.04). When TNM stages I, II and III were combined, the resulting grouping (stage I–III *vs* IV) had am impact on survival, which was similar to that of the extent of disease (81 *vs* 31% 1-year survival, *P*<0.01).

Of the various multivariate models of survival applied, the one selected was chosen because of its clinical relevance, completeness of the data with regard to the included variables and a good statistical fit. According to this model, which included age, gender, anatomic location and extent of disease, only the last one retained statistical significance, with a hazard ratio of 4.0 (95% CI 2.0–8.2, *P*<0.01). When the extent of disease according to VALSG staging system was replaced, in the same model, by TNM stage, this also retained significance, although its *P*-value was higher (*P*=0.03). It is of note that in neither of these models performance status and weight loss were included, due to the high percentage of missing data. However, models that included them, with smaller numbers of available patients, did suggest independent prognostic impact for these two factors (data not shown).

### Influence of specific site

In order to examine the importance of the specific site from which SmCC of the GIT arise, tumours were divided into three groups according to their anatomic location: upper GIT (oesophagus and stomach), lower GIT (large bowel and anus) and other (gallbladder, pancreas and small bowel). As seen in Table 4

**Table 4:** Comparison between SmCC arising at different GI sites (*n*=64)

	**Upper GIT (*n*=24)**	**Lower GIT (*n*=27)**	**Other (*n*=13)**
Median age (years)	65	55	59
Male gender	17/24 (71)	9/27 (33)	5/13 (38)
Second malignancies	6/22 (27)	1/22 (5)	4/10 (40)
Mixed histology	9/23 (39)	10/27 (37)	4/12 (33)
Extensive disease	10/23 (43)	13/27 (48)	7/13 (54)
Response to chemotherapy	3/8 (37)	3/9 (33)	2/5 (40)
Progression rate	18/21 (86)	20/21 (95)	10/13 (77)

GIT: gastrointestinal tract; SmCC=small-cell carcinoma.

a Tumours were grouped into three categories: upper GIT (oesophagus, GEJ and stomach), lower GIT (large bowel and anus) and other (hepatobiliary system and small bowel).

, most of the clinicopathological features of these groups were very similar, including the proportion of mixed histology and ED, response rate to chemotherapy and incidence of progression. In addition, on univariate analysis, no influence of anatomic location on survival was identified. The only notable difference between the groups was in their profile of predisposing medical conditions, as described above.

## DISCUSSION

Small-cell carcinoma of the GIT is a very rare malignancy. Knowledge of this disease is derived predominantly from small series that are all organ specific (e.g. SmCC of the oesophagus) and from extrapolations from what is perceived as an almost identical entity, primary SmCC of the lung. We recently reviewed the literature with regard to SmCC of the GIT. We noted a paucity of information on the disease, and wondered if the commonly held view with regard to its similarity with SmCC of the lung was valid (Brenner *et al*, 2004). In order to expand the available databases, we conducted the current study, trying to exploit a combination of a large database and a somewhat different approach; to the best of our knowledge, this is the first study of this disease focusing on these tumours throughout the GIT and not on the individual location within the GIT from which they arise.

The present study largely confirmed previous concepts of the epidemiology and clinical presentation of the disease. These include its rarity (Remick *et al*, 1987; Acea Nebril *et al*, 1996), the predominance of older patients (Medgyesy *et al*, 2000; Maitra *et al*, 2001), the characteristic presentation (Wick *et al*, 1987; Hoff and Pazdur, 1999), including occasional symptoms that are secondary to hormone production (Taniguchi *et al*, 1973; Chejfec and Gould, 1977; Doherty *et al*, 1984) and the typical advanced stage at diagnosis (Matsui *et al*, 1991; Bennouna *et al*, 2000). The high proportion of oesophageal tumours is also in agreement with previous reports (Ibrahim *et al*, 1984; Remick *et al*, 1987); 30% of our patients and half of the cases reported in the literature had oesophageal tumours. In concordance with the literature, the colon and rectum was the leading location in our study, involved in 39% of the patients (Hoff and Pazdur, 1999). While we observed a nearly equal gender distribution, there seems to be a trend in the literature toward a male predominance in this disease (Medgyesy *et al*, 2000; Madroszyk *et al*, 2001). Other epidemiological features of our patient population, the high prevalence of metachronous cancers and family history of cancer, were not reported previously.

At present, the pathogenesis of SmCC of the GIT is obscure. A leading hypothesis is that these tumours derive from a pluripotent stem cell, which can also give rise to adeno and squamous cell carcinomas. (Ho *et al*, 1984) Two findings in our study, confirming previous reports, seem to be in support of this theory. First, the high proportion of tumours with elements of adeno or squamous cell carcinoma (Craig *et al*, 1995; Maitra *et al*, 2001). Second, the high frequency of medical conditions, which are associated with increased risk for non-SmCC tumours in the same organ (Burke *et al*, 1991; Chetty *et al*, 1993; Yaziji and Broghamer, 1996; Lam *et al*, 2000). The high prevalence of mixed tumour histology may influence treatment. Current chemotherapeutic agents almost invariably fail to eliminate tumour cells of adeno or squamous phenotype completely. Hence, it is possible that in the presence of complete clinical response to chemotherapy, the recurrent tumour will frequently consist of the non-SmCC clones. For patients with LD, this implies a potential role for local therapies.

Small-cell carcinoma of the GIT has been associated with a dismal prognosis. The median survival was described in the range of 6–12 months or of several weeks, for treated or untreated patients, respectively (Redman and Pazdur, 1987; Wick *et al*, 1987; O'Byrne *et al*, 1997). In our study, all but two patients were treated; the median survival was 11 months and 2-year survival was 23%. Similar to others, we also observed a small group (6%) of long-term survivors. The pattern of spread we noted partially concurs with prior reports: while hepatic and nodal involvements were also very frequent in our study, peritoneal and soft-tissue metastases seemed to have been more common than described previously (Matsui *et al*, 1991; Nishimaki *et al*, 1997; Hoff and Pazdur, 1999).

Extensive disease is almost invariably treated by systemic chemotherapy (Nichols and Kelsen, 1989; Huncharek and Muscat, 1995). In contrast, the treatment approach for LD, a potentially curable condition, is presently neither consistent nor uniform; while some authors used only local therapies, mostly surgery and occasionally radiotherapy, others advocated the use of chemotherapy, even alone, in these patients (Kelsen *et al*, 1980; Arai and Matsuda, 1998). Primary treatment of LD in our study varied. With small absolute numbers, this study suggests a potential role for surgery for LD; half of the operated patients retained locoregional control, and four of the six long-term survivors had surgery; two of them with no other treatment. Our study also supports the effectiveness of chemotherapy on survival in this disease; three of the long-term survivors received chemotherapy too. Six patients were treated by a combination of surgery and chemotherapy. Two of these had no evidence of disease for over 7 years and locoregional control was preserved in three. At present, in the absence of data derived from prospective clinical trials, it seems reasonable to treat patients with LD with pre- or postoperative chemotherapy.

The chemotherapy regimens used in our institution were mostly ones used at that time to treat pulmonary SmCC. A 50% response rate was noted for platinum-based and CAV regimens, lower than the 70–90% rate described before (Redman and Pazdur, 1987; Huncharek and Muscat, 1995; Bennouna *et al*, 2000). Whether this discrepancy reflects the fact that most previously reported treatment results derived from studies on oesophageal SmCC or the larger number of patients in our study, the current rate may represent more realistically the impact of chemotherapy in this disease. Compared with pulmonary SmCC, the apparently lower response rate to chemotherapy in GI SmCC may reflect the suggested higher rate of mixed tumour histology in the latter. In any case, from our study and others, cisplatin-based combinations can be presently viewed as the chemotherapeutic regimens of choice in GI SmCC (Nichols and Kelsen, 1989; Hoff and Pazdur, 1999). One should consider the frequent mixed tumour histology in this disease. Of the various options, a combination of cisplatin and irinotecan can potentially provide significant activity against both components. This regimen was shown to be superior to cisplatin/etoposide in pulmonary SmCC (Noda *et al*, 2002) and to be very active in gastro-oesophageal adenocarcinoma (Enzinger *et al*, 1998). Moreover, irinotecan is now considered one of the two most effective agents against colorectal cancer (Rougier and Mitry, 2001). Recently, a response of cisplatin-resistant gastrointestinal SmCC to irinotecan was reported (Sairenji *et al*, 2001).

The current study has identified two prognostic factors in GI SmCC, the extent of disease according to the VALSG classification and TNM stage, and the prognostic impact of two others, performance status and weight loss, was implied. While preliminary, our study seems to suggest that clear prognostic stratification exists only between TNM stages I–III and stage IV. If confirmed, this may lead to the acceptance of the simpler VALSG system as the primary staging method for GI SmCC, similar to pulmonary SmCC. In the only prognostic factor analysis in that disease published to date, Casas *et al* (1997) also demonstrated the prognostic impact of the extent of disease. In their study, involving 199 previously reported patients with oesophageal SmCC, the median survival of patients with LD and ED was 8 and 3 months, respectively (*P*<0.01) (Casas *et al*, 1997). The prognostic influence of both the extent of disease and performance status has also been demonstrated in primary SmCC of the lung (Radford *et al*, 1992; Lassen *et al*, 1999; Paesmans *et al*, 2000; Nicholson *et al*, 2002).

Our study may offer an opportunity to test the widely accepted hypothesis that SmCC of the GIT and the lung are nearly identical. Outlined in Table 5

**Table 5:** Comparison of features from the current study with those of SmCC of the lung

**Similarities**	**Dissimilarities**
Neuroendocrine features, with occasional symptomatic hormone production	Smaller proportion of smokers (45 *vs* 90% in SmCLC^a^)
High metastatic potential	Presence of predisposing medical conditions for non-SmCC tumours in the same organ
Pattern of spread	Higher rate of non-SmCC components (37 *vs* 1–3% in most SmCLC series)
Typical overall chemosensitivity	Suggested higher proportion of LD at presentation (52 *vs* 30–40% in SmCLC)
Overall dismal prognosis	Larger proportion of long-term survivors following surgery (4 of 17 *vs* 1% in SmCLC)
Prognostic impact of extent of disease and performance status	Suggested lower efficacy of radiotherapy in preserving LRC (14% *vs* 70% in SmCLC)
	Lower response rate to CAV and platinum-based chemotherapy (50 *vs* 80–90% (LD) and 60–80% (ED) in SmCLC)

LRC=locoregional control; CAV=cyclophosphamide, doxorubicin and either vincristine or etoposide; LD=limited disease; ED=extensive disease; SmCC=small-cell carcinoma.

a Accepted figures in the literature for SmCC of the lung (SmCLC).

are representative differences and similarities between findings from our study and known features of primary lung SmCC. Despite an extensive overlap, the comparison seems to imply some differences between the two entities. Of importance, these include a differential efficacy of various treatment modalities, such as a role for surgery in GI SmCC. Both the disparities and similarities identified here were suggested before (Nichols and Kelsen, 1989; Matsui *et al*, 1991; Nishimaki *et al*, 1997). Moreover, initial reports seem to imply only partial overlap between the molecular biology patterns of the two entities (Takubo *et al*, 1999; Lam *et al*, 2000; Maitra *et al*, 2001). This suggests the need for further investigation, and the drawback of managing SmCC of the GIT totally on the basis of extrapolations from lung SmCC.

In summary, the present study suggests that SmCC arising from various locations within the GIT can be viewed and treated as a one clinical entity. We noted the prevalence of mixed tumour histology, the value of the VALSG staging system and the potential role of surgery, combined with chemotherapy, in the treatment of LD. Nonetheless, more data are required before any solid clinical guidelines are established. Since prospective clinical trials are unlikely in this rare disease, large retrospective surveys, such as ours, and correlative studies, represent the main source of this information.
